# Strength training versus robot-assisted gait training after incomplete spinal cord injury: a randomized pilot study in patients depending on walking assistance

**DOI:** 10.1186/1743-0003-11-4

**Published:** 2014-01-09

**Authors:** Rob Labruyère, Hubertus J A van Hedel

**Affiliations:** 1 Spinal Cord Injury Center, Balgrist University Hospital, Zurich, Switzerland; 2 Institute of Human Movement Sciences and Sport, ETH Zurich, Switzerland; 3 Pediatric Rehab Research Group, Rehabilitation Center, University Children’s Hospital Zurich, Affoltern am Albis, Switzerland

**Keywords:** Rehabilitation, Spinal lesion, Training study, Paraplegia, Lokomat, Randomized clinical trial, Walking, Pain

## Abstract

**Background:**

Task-specific locomotor training has been promoted to improve walking-related outcome after incomplete spinal cord injury (iSCI). However, there is also evidence that lower extremity strength training might lead to such improvements. The aim of this randomized cross-over pilot study was to compare changes in a broad spectrum of walking-related outcome measures and pain between robot-assisted gait training (RAGT) and strength training in patients with chronic iSCI, who depended on walking assistance. We hypothesized that task-specific locomotor training would result in better improvements compared to strength training.

**Methods:**

Nine participants with a chronic iSCI were randomized to group 1 or 2. Group 1 received 16 sessions of RAGT (45 min each) within 4 weeks followed by 16 sessions of strength training (45 min each) within 4 weeks. Group 2 received the same interventions in reversed order. Main outcome measures were the 10 m Walk Test (10MWT) at preferred and maximal speed. Furthermore, we assessed several measures such as walking speed under different conditions, balance, strength, and 2 questionnaires that evaluate risk of falling and pain. Data were collected at baseline, between interventions after 4 weeks, directly after the interventions and at follow-up 6 months after the interventions. Pain was assessed repeatedly throughout the study.

**Results:**

There were no significant differences in changes in scores between the 2 interventions, except for maximal walking speed (10MWT), which improved significantly more after strength training than after RAGT. Pain reduced after both interventions.

**Conclusion:**

In patients with chronic iSCI dependent on walking assistance, RAGT was not more effective in improving walking-related outcome compared to lower extremity strength training. However, the low sample size limits generalizability and precision of data interpretation.

**Trial registration:**

This study was registered at Clinicaltrials.gov (NCT01087918).

## Introduction

In a recent *Point of View*, it was highlighted that neither body weight-supported treadmill training (BWSTT) nor robot-assisted gait training (RAGT) have fulfilled the high expectations that were raised by researchers and clinicians in the field of neurorehabilitation [1]. There is no evidence yet that these methods are superior to over-ground training to improve ambulation in neurological patients, even if they were thought to have a large potential to advance gait rehabilitation [2,3].

Nevertheless, driven gait orthoses like the Lokomat (Hocoma AG, Volketswil, Switzerland) [4,5] have been introduced in numerous rehabilitation settings to treat patients with locomotor dysfunction [6]. A trend has been described that robot-assisted gait training (RAGT) might even replace rather than complement conventional physiotherapeutic interventions [7]. This is undesirable as superiority of task-specific locomotor training - meaning training the task we want the patients to get better at (in this case walking) - has not been proven sufficiently. Additionally, recent studies and reviews have also shown the limitations of RAGT compared to BWSTT in different patient populations [1,8,9].

After years of promoting task-specific training combined with the popularity of applying BWSTT, it seems logical that task-specific locomotor training, such as RAGT, might improve ambulatory function more than lower extremity muscle strength training. One of the main reasons therefor is that task-specific training provides locomotion-relevant afferent input to spinal central circuitries that generate rhythmic stepping behavior [10]. While such input is not provided during strength training of the lower limbs (without training walking), improvements in ambulatory function have been shown after stroke or incomplete spinal cord injury (iSCI), since locomotor capacity correlates well with strength of leg muscles, like hip flexors or extensors [11,12]. Accordingly, data obtained from 3 patients with iSCI suggested that a 12-week resistance and plyometric training program (plyometric exercises consist of powerful movements where muscles are rapidly and repeatedly stretched and contracted) led to increased walking speeds in the 10 m Walk Test (10MWT) at preferred and maximal speed [13].

While task-specific locomotor training appeared superior over strength training for a long time in patients with stroke [14,15], the LEAPS trial recently concluded that a home-exercise program, with the aim of enhancing flexibility, strength, coordination or balance, was equivalent to locomotor training [16]. Recent studies also showed that after iSCI, complex muscle coordination and motor programs appear intact while muscle strength is affected [17,18]. This is different compared to stroke, where complex muscle coordination was disturbed even in the “unaffected” leg [18]. However, these studies investigated single joint movement tasks, and it is unclear whether these findings can be translated into more functional movements like walking. Therefore, the primary aim of this study was to compare gait-related outcomes of lower extremity strength training and task-specific locomotor training in patients with chronic iSCI, especially since recent publications failed to demonstrate superiority of one intervention over another one [19-21]. Additionally, as many patients with spinal cord injury experience chronic pain [22] and already single sessions of BWSTT can lead to pain reduction [23], we investigated the immediate and longitudinal effects of both interventions on pain intensity.

## Methods

### Design overview

This study was a randomized cross-over open label clinical pilot study. The main outcome measures were videotaped and separately scored by a blinded assessor.

### Setting and participants

Inclusion criteria were: aged 18-70 years, chronic iSCI (time after injury >1 y) and sensorimotor incomplete (grade C or D on the American Spinal Cord Injury Association [ASIA] Impairment Scale [AIS] [24]). The motor level of the lesion was between C4 and T11 to exclude patients with peripheral lesions. Furthermore, participants had to walk with at most moderate assistance at the time of inclusion (i.e. a score of <6 in the “mobility outdoors” item of the Spinal Cord Independence Measure [SCIM] [25]). Cognitive capacity to follow verbal instructions was tested with the Mini Mental State Examination (required score: ≥26) [26].

Participants were excluded if they presented contra-indications for training in the Lokomat system (according to the manual), had injuries limiting training, as well as orthopedic, psychiatric or neurological diseases, except for the iSCI.

**Figure 1:**
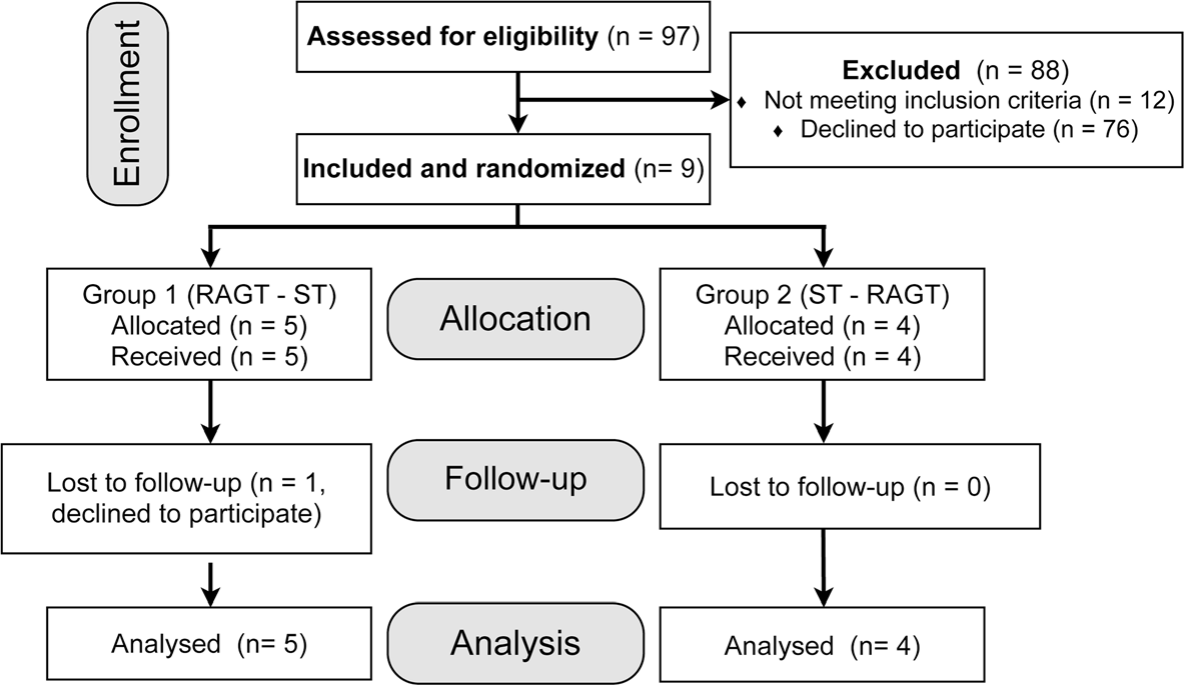
**Consolidated standards of reporting trials diagram.** Abbreviations: RAGT = robot-assisted gait training; ST = strength training.

Participant enrollment started in March 2009, and the final participant completed training in April 2011. We intended to include as many participants as possible. The research database of our hospital was screened for possible candidates. A total of 97 entries were found to approximately match inclusion and exclusion criteria. These patients were contacted and invited. Out of 10 patients who were screened on site, 1 did not meet the inclusion criteria. The other 9 (all AIS D) participated and completed the intervention. Figure 1 shows the CONSORT (Consolidated Standards of Reporting Trials) diagram. Characteristics of the participants are displayed in Table 1. Participants were asked to maintain their regular medication scheme and inform the principal investigator about any changes or extraordinary events. Two participants (P03&P05) wore ankle-foot orthoses, and these were kept on during all training sessions and assessments.

**Table 1:** **Subjects**’ **characteristics**

**ID**	**IG**	**Age (years)**	**Height (cm)**	**Weight (kg)**	**Sex**	**Months p. injury**	**Level of lesion**	**Etiology**	**WISCI**	**CA**	**Pain**
P01	2	69	178	68	m	16	C4	Trauma	13	Yes	npb
P02	1	69	178	80	m	13	T8	Tumor	16	Yes	None
P03	1	43	163	54	f	84	T11	Trauma	12	Yes	nap & nbp
P04	2	60	166	75	f	21	T4	Abscess	16	Yes	nap & ms
P05	1	60	179	92	m	44	T11	Herniation	9	No	ms
P06	2	41	161	48	f	189	C6	Trauma	16	Yes	nbp & ms
P07	1	53	183	85	m	29	C5	Herniation	13	Yes	nap & nbp
P08	2	67	164	89	f	27	C5	Herniation	16	Yes	nap & nbp
P09	1	69	179	93	m	26	C4	Trauma	16	Yes	nbp & ms
Mean ± SD		59 ± 11	172 ± 9	76 ± 16		50 ± 56			14 ± 3		
Median		60	178	80		27			16		

Abbreviations: *AIS* ASIA Impairment Scale, *C* cervical, *CA* community ambulator, *f* female, *ID* identification, *IG* intervention group, *m* male, *ms* musculoskeletal pain, *nap* neuropathic at level pain, *nbp* neuropathic below level pain, *p* post, *T* thoracic, *WISCI* Walking Index for Spinal Cord Injury.

All training sessions and assessments took place at the spinal cord injury center of our hospital. This study was registered at http://www.clinicaltrials.gov (NCT01087918).

### Ethics statement

All participants gave written informed consent to participate and to have their anonymized data published, and the study protocol was approved by the ethics committee of the Canton of Zurich, Switzerland. The study was conducted according to the principles expressed in the Declaration of Helsinki.

### Randomization and interventions

The aim of the study was to compare RAGT with strength training in patients with chronic iSCI. Randomization for group assignment was performed by dice-rolling before pretests were performed. Group 1 received 16 sessions of RAGT within 4 weeks followed by 16 sessions of strength training within 4 weeks. Group 2 received the same intervention in reversed order.

RAGT was performed with the Lokomat System (for details see references [4,5]). However, in the present study, we used customized software that, besides the position-controlled mode (standard Lokomat software), had 2 additional modes. In position-controlled mode, the end point of the robotic leg is exactly defined for each particular time point during the gait cycle. In path control mode, however, there is a virtual tunnel around the preprogrammed gait trajectory and within this tunnel, the participant can freely move his leg. If the participant’s leg deviates from this virtual tunnel, the Lokomat pushes the leg back into the tunnel [27,28]. With this control mode, the participant trains a more functional gait pattern that better represents “over ground walking” compared to the standard position-controlled mode. Path control mode allows individual variability within the gait cycle, which is relevant for motor learning. For the most difficult training mode, we added treadmill speed control to the path control mode. Here, participants additionally could control the speed of the belt with their body posture. By slightly moving their trunk forward (increase in speed) or backward (decrease in speed), they could control treadmill belt speed within a defined range. These modes enabled us matching training intensity to the participants’ capabilities and guaranteed active participation, whereas in earlier clinical trials with the conventional system, theoretically, participants could just be passive [21,29]. Speed was limited to 4.0 km/h (=1.11 m/s) in every mode (average training speed in these patients usually is around 1.5-2.0 km/h).

Training duration per session was 45 min for both interventions (actual training time, including maximally 2 breaks of 1-2 min during RAGT, and including warming-up during strength training and breaks to change from one exercise to the next). All trainings were executed under the supervision of a movement scientist. The first session of RAGT focused on adjusting the system to the participant. To allow participants to familiarize themselves with the system, training started with approximately 30% bodyweight support and a treadmill speed of 1-2 km/h. All participants initially started training using foot lifters to ensure foot clearance during swing phase. If control and strength of ankle dorsiflexion improved, the tension of the foot lifters was decreased until active dorsiflexion was sufficient to remove the foot lifters. In subsequent sessions, training intensity was increased progressively by changing walking speed, level of bodyweight support, robotic support or by applying the next higher training mode. Modes were easily switchable; therefore, different training modes were not applied exclusively, but rather alternately, where time spent training in the more difficult mode steadily increased according to the participant’s capabilities. All but one participant (P05, see Table 1) trained in interactive mode most of the time and 2 participants managed to walk in interactive mode with control of treadmill speed (P02&P04). The amount of body-weight support was adjusted individually to achieve adequate knee extension during stance phase and toe clearance during swing phase.

Strength training was aimed particularly at lower limb muscles without performing task-specific walking exercises. The first session of strength training focused on the configuration of a set of feasible exercises, and this set was adapted throughout the intervention period to meet the participant’s needs, capabilities and progress. One training session consisted of 10 min of warming up on a bicycle, rowing ergometer or cross trainer, followed by approximately 4-6 exercises, where we aimed for 3 bouts of 10-12 repetitions at 70% of maximal voluntary contraction, which was determined in the first session. These exercises were put together individually; examples of exercises that were performed by all participants were isotonic leg press in supine position and isotonic hip adduction, abduction, flexion and extension at the wall pulley (standing, with/without resistance) or on the examination table (lying, with/without additional weights).

### Outcomes and follow-up

**Figure 2:**
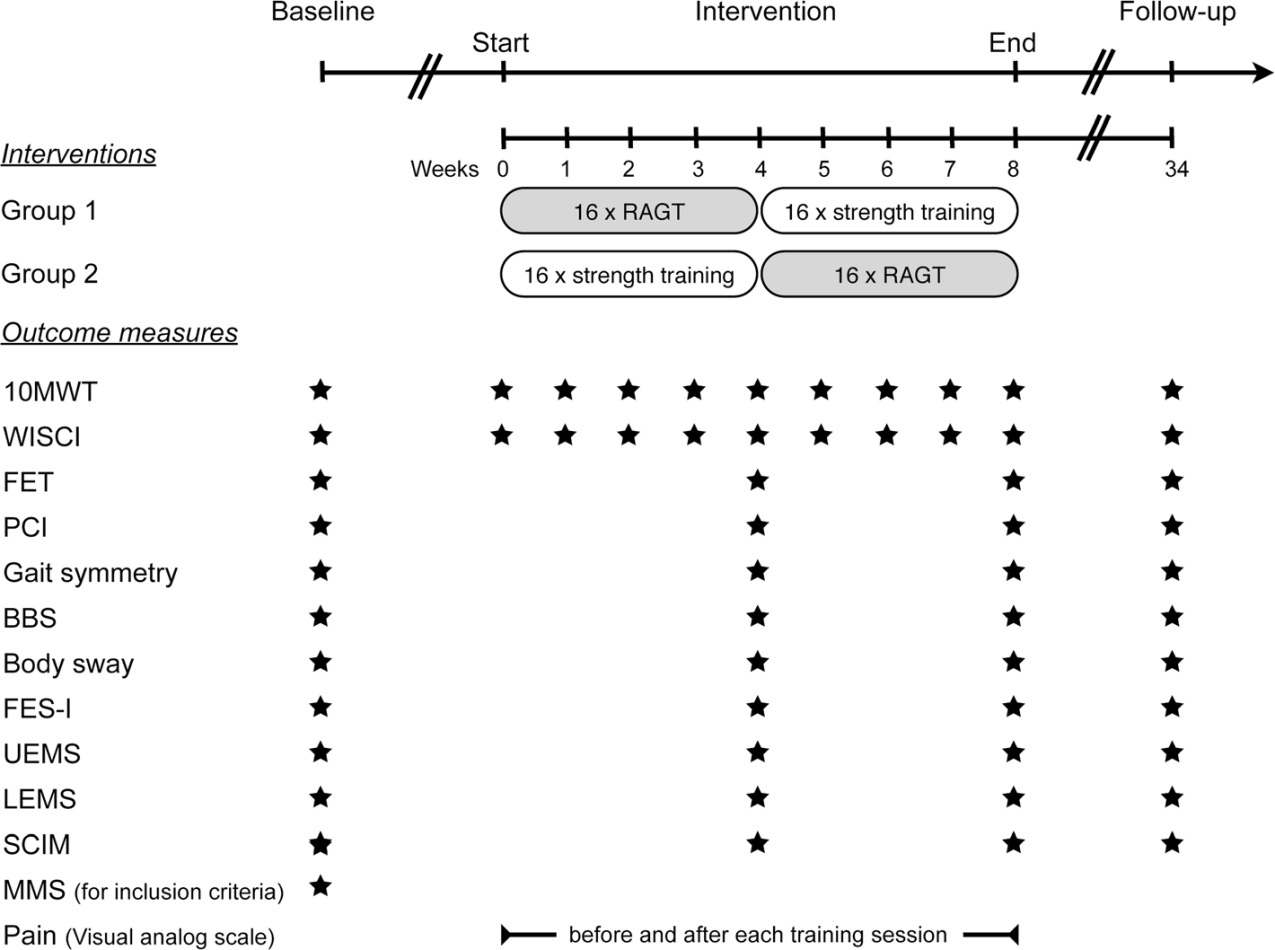
**Application scheme of outcome measures.** Abbreviations: 10MWT = 10 m Walk Test; BBS = Berg Balance Scale; FES-I = Falls Efficacy Scale – International Version; FET = Figure Eight Test; LEMS = lower extremity motor score; MMS = Mini Mental State Examination; PCI = Physiological Cost Index; RAGT = robot-assisted gait training; SCIM = Spinal Cord Independence Measure; UEMS = upper extremity motor score; WISCI = Walking Index for Spinal Cord Injury.

We chose the 10MWT at preferred and maximal speed as primary outcome measures. Additionally, on a hypothesis-generating basis, we explored a broad range of secondary outcome measures and we investigated the longitudinal course of general pain by assessing it before and after each training session. All outcome measures were applied according to Figure 2. Follow-up assessments were performed to investigate the sustainability of potentially induced changes in outcomes. Given the cross-over nature of this study, it was not possible to make statements about sustainability for each intervention specifically.

#### Primary outcome measures

Walking speed was evaluated with the 10MWT [30]. Participants were instructed to walk at their preferred and maximal speed, but were not given verbal encouragement. The 10MWT was performed with a “flying start” (while participants walked about 14 m, the time was measured for the intermediate 10 m) [31]. Results were converted to walking speed (m/s).

#### Secondary outcome measures

##### General

Selected items of the International Spinal Cord Injury Core Data Set [32] were collected (demographic and iSCI-specific data of interest, Table 1), and the motor score of the neurological examination according to the ASIA International Standards [24] was evaluated (upper extremity motor score [UEMS] in participants with tetraplegia only, lower extremity motor score [LEMS] in all participants). Furthermore, we applied the SCIM to assess independence and evaluated several outcome measures of walking and balance.

##### Walking

We applied the WISCI [33] to assess which kind of assistive devices or personal assistance the participants needed to cover 10 m.

To test the participants’ ability to adapt their gait to several circumstances, we applied the Figure Eight Test (FET), which was previously described in more detail and shown to be valid [34]. The test required participants to traverse a 10 m long figure of eight-shaped track 6 times, each time under a different condition, including:

–FET preferred: at preferred walking speed.

–FET maximal: at maximal safe walking speed.

–FET vision: subjects wore vision-blurring glasses.

–FET obstacle: Two obstacles, one in each curve, had to be overstepped.

–FET foam: Subjects wore foamed soles under their shoes.

–FET dual task: During walking, a list of questions had to be answered as quickly as possible.

The time needed for each condition was recorded and converted to walking speed (m/s). Except for FET maximal, participants were directed to walk at self-selected speed corresponding to their preferred comfortable walking speed in everyday life.

We estimated energy expenditure with the Physiological Cost Index (PCI) [35]. The PCI was assessed on a treadmill. First, participants stood still for 2 min and the mean heart rate of the last 10 s was used as heart rate at rest. Then they walked for 3 min at the same speed as previously determined in their first 10MWT at preferred speed. The PCI was calculated as follows: PCI = (steady-state heart rate – heart rate at rest)/ambulatory velocity.

We assessed symmetry of gait, which has been shown to improve after locomotor-specific training in patients with iSCI [20]. It is an important marker for the quality of gait and an accurate indicator of changes in the walking pattern, even on a sub-clinical level [36]. There is evidence that gait symmetry also improves after lower extremity strength training in patients with stroke [37]. Gait symmetry was measured by comparing lengths of stance and swing phases of each single leg (by dividing stance time [in % of whole step] right by stance time left) with portable insoles. If the value was >1, it was inverted to ensure comparability. Gait symmetry was measured in 8 participants only, due to infrastructural issues.

##### Balance

To cover the risk of falling, which is increased up to 75% after iSCI [38,39], we measured balance, which is considered important for functional ambulation after iSCI [40]. Barbeau and Visintin showed that manual BWSTT improves balance in patients with stroke [41]. However, as participants are strongly fixated in the robot, it remains questionable whether balance becomes trained during RAGT, as recently discussed for patients with stroke [9,42]. As a performance-based measure of balance, we used the Berg Balance Scale (BBS) [43]. As a marker of static balance, we measured the maximal mediolateral amplitude of the center of pressure movement over 30 s on a force plate (Kistler Instrumente AG, Winterthur, Switzerland). Participants were asked to stand as still as possible and to fixate a given object with their eyes. The distance between their feet was 10 cm. The test was done twice, and the best try counted. To assess fear of falling while performing different activities of daily living, we applied the international version of the Falls Efficacy Scale (FES-I) [44].

##### Pain

To assess the influence of the interventions on general pain intensity, participants were asked to rate their current pain immediately before and 5 min after each training session on a 100 mm visual analog scale (VAS) that was confined by the terms “no pain” on the left side (0%) and “unbearable pain” (100%) on the right side. The instructions were: “Please rate the general pain you are experiencing at this moment”. To avoid the influence of circadian pain patterns, trainings were always performed on the same time of the day. One participant did not suffer from pain at all, and, therefore, pain assessments were done in 8 participants.

### Statistical analyses

**Figure 3:**
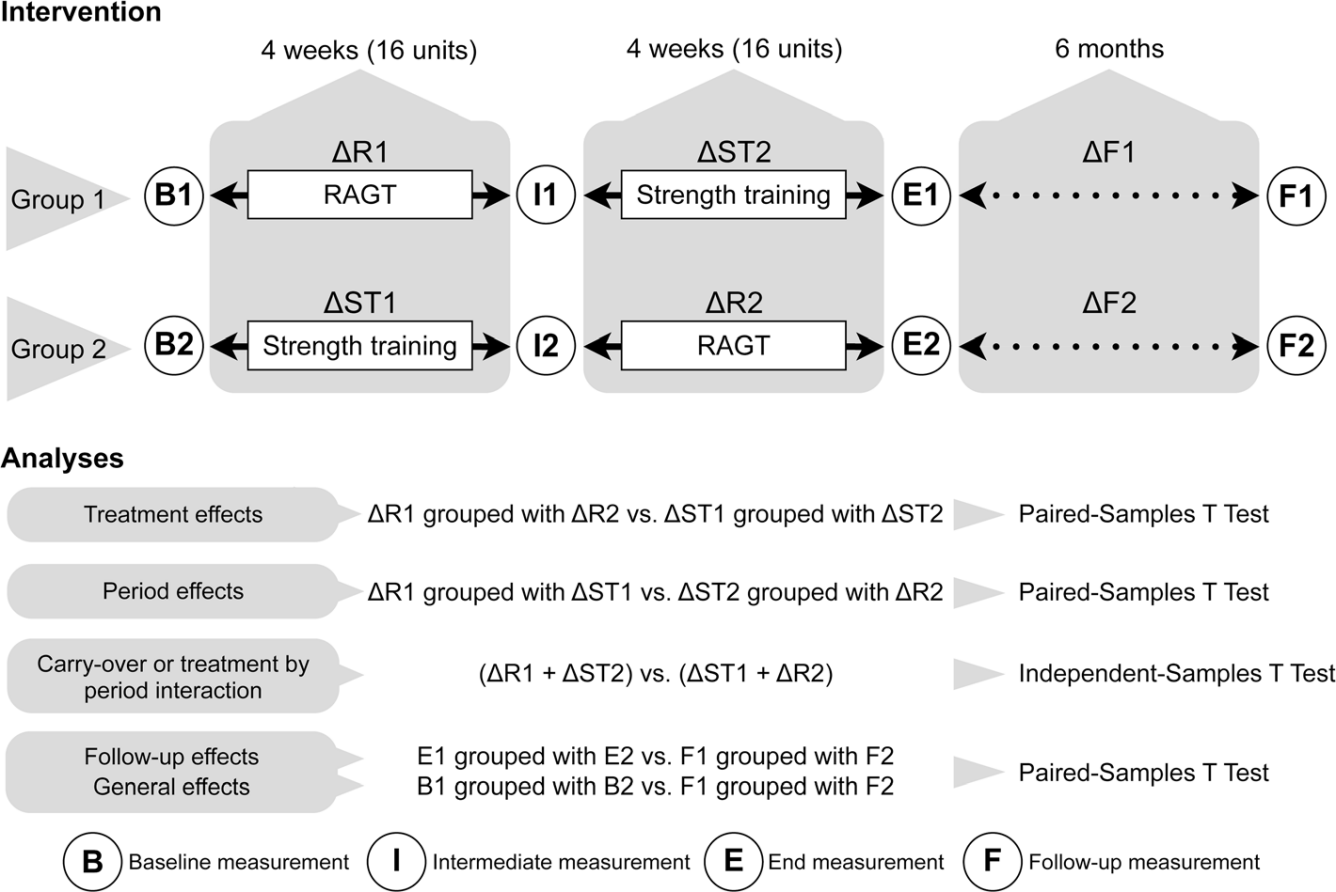
**Overview of the protocol and the statistical methods applied.** Pain assessments are not included in this figure, as they were not performed at the indicated measurements but during the interventions themselves. Abbreviations: RAGT = robot-assisted gait training; ΔF1 = improvement during follow-up in group 1; ΔF2 = improvement during follow-up in group 2; ΔR1 = improvement during robot-assisted gait training in group 1; ΔR2 = improvement during robot-assisted gait training in group 2; ΔST1 = improvement during strength training in group 1; ΔST2 = improvement during strength training in group 2.

Given our interest in the impact of 2 training interventions on different outcomes in patients with iSCI, statistical analyses were applied to quantify differences in change scores between the two interventions for each outcome. The use of a cross-over design was chosen to reduce the impact of inter-individual variability by having each participant act as its own control [45]. To allow an upfront interpretation, we adopted parametric testing, as only a few secondary outcome measures did not show normally distributed within-subject change scores (tested with the Shapiro-Wilk Test), namely SCIM, WISCI, UEMS and LEMS. This approach allows including the data in future meta-analyses. Figure 3 displays an overview of the applied statistical analyses, accounting for the characteristics of cross-over designs. As carry-over and treatment by period interaction are unlikely to be separable [45], we did one analysis for both combined.

To evaluate the longitudinal influence of the interventions on pain intensity, we plotted the mean VAS-scores before and after training against time for all 16 sessions for each intervention and performed linear regression analyses. We considered a longitudinal decrease in pain intensity to be significant, when the regression coefficient was significantly smaller than zero. Additionally, we investigated the short-term effect of training on pain intensity by subtracting the mean VAS-scores after training from those before training for each intervention and compared these change scores with a Paired-Samples T Test.

For all outcome measures, intention-to-treat analysis was performed using the last observation carried forward method to account for missing data. Only 1 participant refused to take part in the follow-up measurement, and this was treated as missing data. Alpha was set at 0.05.

## Results

All participants completed both interventions and no adverse events occurred. Age and time since injury were comparable in both groups. During RAGT, participants walked on average 1731 ± 265 m per session. During strength training, mean resistance was increased in all the exercises that were performed by all participants. For leg press with both legs, mean increase of resistance from the first to the last session was 17 ± 11 kg. Also for one-legged exercises, resistance could be increased (hip adduction: 4 ± 2 kg, hip abduction: 2 ± 1 kg, hip flexion: 3 ± 1 kg and hip extension: 3 ± 2 kg).

### Treatment effects

#### Main outcome measures

**Figure 4:**
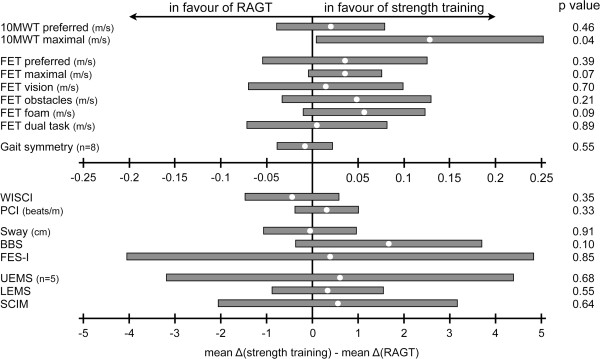
**Overview of treatment effects on changes in scores of all outcome measures.** The white dots correspond to the mean difference between mean change score due to strength training and mean change score due to robot-assisted gait training in raw units and the grey bars depict the associated 95% confidence interval. P-values ≤ 0.05 are bold. Abbreviations: 10MWT = 10 m Walk Test; BBS = Berg Balance Scale; FES-I = Falls Efficacy Scale – International Version; FET = Figure Eight Test; LEMS = lower extremity motor score; PCI = Physiological Cost Index; RAGT = robot-assisted gait training; SCIM = Spinal Cord Independence Measure; UEMS = upper extremity motor score; WISCI = Walking Index for Spinal Cord Injury.

Improvements of changes in scores of the 10MWT at maximal speed were larger due to strength training compared to RAGT (Figure 4 based on onset and end data from Table 2). There was no statistical difference between the interventions with respect to the changes in scores of the 10MWT at preferred speed (Figure 4).

**Table 2:** **Mean values** ± **standard deviations of all participants**

**Outcome measure**	**Onset RAGT**	**End RAGT**	**Onset strength training**	**End strength training**	**Follow-up**^**c**^
10MWT preferred (m/s)	0.62 ± 0.23	0.66 ± 0.29	0.58 ± 0.19	0.64 ± 0.23*	0.66 ± 0.26
10MWT maximal (m/s)	0.79 ± 0.31	0.80 ± 0.35	0.66 ± 022	0.80 ± 0.28*	0.78 ± 0.28
FET preferred (m/s)	0.52 ± 0.20	0.53 ± 0.20	0.50 ± 0.16	0.54 ± 0.21	0.53 ± 0.18
FET maximal (m/s)	0.62 ± 0.21	0.63 ± 0.23	0.60 ± 0.20	0.65 ± 0.22*	0.64 ± 0.22
FET vision (m/s)	0.49 ± 0.21	0.50 ± 0.21	0.48 ± 0.20	0.50 ± 0.20	0.43 ± 0.17
FET obstacle (m/s)	0.42 ± 0.19	0.41 ± 0.20	0.39 ± 0.19	0.43 ± 0.20*	0.40 ± 0.20
FET foam (m/s)	0.42 ± 0.19	0.42 ± 0.20	0.39 ± 0.18	0.45 ± 0.20*	0.41 ± 0.13
FET dual task (m/s)	0.45 ± 0.19	0.48 ± 0.18	0.44 ± 0.15	0.48 ± 0.18	0.43 ± 0.17
Gait symmetry^a^	0.91 ± 0.18	0.93 ± 0.13	0.93 ± 0.13	0.96 ± 0.09	0.92 ± 0.09
WISCI	14.1 ± 2.5	14.9 ± 3.1	14.4 ± 2.6	14.8 ± 2.9	15.5 ± 2.7
PCI (beats/m)	0.76 ± 0.40	0.88 ± 0.70	0.84 ± 0.74	0.65 ± 0.41	1.04 ± 1.14
Sway (cm)	2.09 ± 1.40	2.13 ± 1.98	2.48 ± 1.88	2.60 ± 2.19	2.66 ± 2.21
BBS	43.3 ± 14.7	44.4 ± 14.7	42.7 ± 14.0	45.4 ± 14.7*	42.9 ± 16.3
FES-I	26.6 ± 8.7	26.4 ± 9.2	25.6 ± 7.3	25.1 ± 5.5	23.8 ± 6.7
UEMS^b^	43.0 ± 2.5	43.4 ± 3.2	42.4 ± 2.1	43.4 ± 2.6	44.3 ± 1.9
LEMS	40.9 ± 7.5	41.6 ± 7.3	40.4 ± 6.6	41.4 ± 6.9*	41.0 ± 7.0
SCIM	88.4 ± 7.9	89.2 ± 7.6	87.9 ± 8.1	89.2 ± 7.9	88.9 ± 6.5

Please note that data were pooled from group 1 and group 2, meaning that e.g. data from onset RAGT cannot directly be compared with data from onset strength training.

*Denotes a significant (p ≤ 0.05) intra-intervention before-after difference as determined by a Paired-Samples T Test. ^a^n = 8 (n = 7 for follow up data). ^b^n = 5 (n = 4 for follow up data). ^c^One participant did not attend follow-up measurements. *Abbreviations:10MWT* 10 m Walk Test, *FET* Figure Eight Test, *WISCI* Walking Index for Spinal Cord Injury, *PCI* Physiological Cost Index, *BBS* Berg Balance Scale, *FES-I* Falls Efficacy Scale – International Version, *UEMS* upper extremity motor score, *LEMS* lower extremity motor score, *SCIM* Spinal Cord Independence Measure.

#### Secondary outcome measures

There were no significant differences in changes in scores between the two interventions for all secondary outcome measures (Figure 4). However, tests like the FET maximal, FET foam and the BBS showed a tendency (0.05 < p-value < 0.10) toward greater improvements after strength training (Figure 4).

### Period and carry-over effects and follow-up results

There was no statistically significant period effect for any outcome measure. Furthermore, we could not find any statistically relevant carry-over/treatment by period interaction. No outcome measure at the end of the second intervention was significantly different from outcome measures at follow-up 6 months later (ranging from p = 0.14 for LEMS to p = 0.93 for 10MWT at preferred speed, results of follow-up measurements are displayed in Table 2). However, there were outcome measures that were better at follow-up compared to baseline (two-sided Paired-Samples T Test; p = 0.05 for 10MWT at preferred speed, p = 0.02 for SCIM and p < 0.01 for LEMS).

### Pain assessments

**Figure 5:**
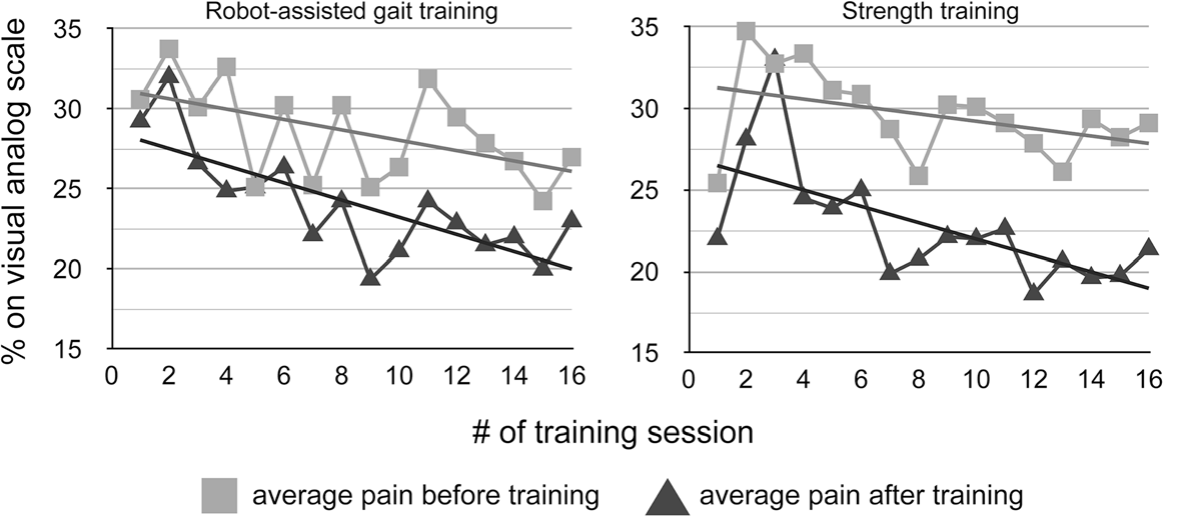
Course of pain rating on a 100 mm visual analog scale from before and after each training session for both interventions.

Overall, 16 sessions of Lokomat training reduced pain intensity over time. The regression coefficient calculated over the mean VAS-scores (before training) was -0.33 (p = 0.04, 95% confidence interval (CI): [-0.02, -0.63], Figure 5). For the pain scores derived after training, the regression coefficient was -0.54 (p < 0.01, 95% CI: [-0.28, -0.80], Figure 5). During the strength training intervention, pain reduced as well, but only significantly after training (before training: regression coefficient = -0.23, p = 0.12, 95% CI: [-0.52, 0.06]; after training: regression coefficient = -0.50, p < 0.01, 95% CI: [-0.17, -0.83], Figure 5).

The interventions caused an immediate reduction of pain that was slightly but significantly larger after strength training (-6.8 ± 2.5%) than after RAGT (-4.5 ± 2.2%, p < 0.01).

## Discussion

The applied outcome measures covered practically all domains of the International Classification of Functioning, Disability and Health [46] on which RAGT or strength training could have an effect. Results of the 10MWT at maximal speed and trends in other outcome measures suggested that strength training was associated with greater improvements compared to RAGT. However, due to low statistical power, a statement of superiority of strength training to RAGT cannot be made. Additionally, clinically meaningful changes (CMC), were rarely reached.

### Walking

Results of the 10MWT already point out that RAGT was not superior compared to strength training. Especially the changes in scores of the 10MWT at maximal speed were better after strength training compared to RAGT. One the one hand, this was unexpected as the walking speed in the Lokomat at the end of the intervention exceeded over-ground walking speed in the 10MWT at maximal speed in 6 of 9 subjects, meaning that we specifically trained fast walking. However, Field-Fote et al. already reported that treadmill speed in RAGT does not seem to be an essential factor for locomotor outcomes in subjects with iSCI [20]. On the other hand, Kim et al. showed that muscle strength correlates with walking speed [12]. Since leg muscle strength improved after strength training (Table 2) this might have led to higher walking speeds. Also, preferred walking speed did not change significantly after RAGT, which is in line with results of studies published earlier [20,29]. However, the main difference to these studies is that, due to the new Lokomat control strategies applied in this study, participants had to contribute actively, thus eliminating passiveness as a reason for the performance of RAGT. Yet, there are also studies where RAGT led to improvements of preferred walking speed [47,48].

Efficacy of RAGT seems to depend on the initial walking function of the participant. Those with lower initial walking speeds are likely to profit more from this intervention compared to those with higher walking speeds [20,47]. Preferred walking speed before intervention was around 0.4 m/s in the study of Wirz et al. [47] and 0.45 m/s in the study of Hornby et al. [48], whereas participants in this study walked considerably faster when they entered the study (0.57 ± 0.20 m/s). Nevertheless, none of the participants reached the speed of 0.7 m/s, which has been described to distinguish between the categories “walker with aid” and “walker without aid” [49].

Figure 4 depicts that strength training could have a greater impact on ambulatory function compared to RAGT in the sampled population. A possible reason might be that predominantly strength is affected in subjects with iSCI, while accurate muscle activation remains largely unaffected [18]. This is also reflected in the consistent improvement in walking speed these subjects experienced after strength training, irrespective of which FET condition we applied (Table 2).

There was no difference between the interventions with respect to PCI. Differences between resting and walking heart rate were very small indicating that walking for 3 min at preferred speed was not a very energy-demanding load in the present sample. Additionally, low walking speeds compared to those of the non-disabled population led to a large statistical data spread. Therefore, the PCI must be looked at critically after iSCI which has been noted earlier [35].

Gait symmetry did not change, and this is in line with results of another study [21]. It must be noted that all but one (P05) subject had no relevant strength differences between their right and left leg.

### Balance

There were no statistical differences between the changes in scores due to RAGT and strength training for balance measures. However, keeping the low statistical power in mind, improvements in the BBS tended to be larger after strength training compared to RAGT. At least the effectiveness of strength training could be anticipated, as it was observed in other populations, such as patients with stroke [50] or healthy elderly [51].

While RAGT improved the BBS score in patients with stroke [9], its effect on static balance has herewith been investigated for the first time. Neither intervention had an influence on static balance. With the current hardware of the Lokomat, one can assume that static balance cannot really be trained; subjects are firmly attached to the device and they are secured from falling by different mechanisms (body weight support, hand rails, foot lifters).

To summarize, it seems that if there is adequate residual muscle strength (to perform walking with some assistance) after iSCI, strength training certainly does not perform worse compared to RAGT when it comes to improving ambulatory function. Further studies are necessary to determine what level of muscle strength (motor score) is needed to recommend strength training (in combination with over-ground gait training) to produce better clinical outcome, as an extension to the work done by Behrman et al. in the field of BWSTT [52]. Nevertheless, RAGT might play an important role during the acute phase, when patients have little residual muscle strength.

### Clinically meaningful changes

To make a statement about the relevance of the differences between both interventions, we compared them with available CMCs from literature. For the 10MWT at preferred speed, we found a CMC of 0.04-0.06 m/s for healthy elderly [53], which lies well within the 95% confidence interval of the between-group difference in the present study (Figure 4). This is not the case for the CMC for patients with Parkinson’s disease, which was estimated to be 0.18 m/s [54]. The latter publication also provides a CMC of 0.25 m/s for the 10MWT at maximal speed, which builds the edge of the 95% confidence interval of the between-group difference in this study.

Furthermore, we found a CMC of 1 point for the WISCI [55], a CMC of 6 points for the BBS [56] and a CMC of 4 points for the SCIM [57]. Of these, only the CMC of the WISCI lies within the 95% confidence interval of the difference between interventions.

### Period and carry-over effects and follow-up results

Statistical analyses did not reveal significant period effects. Against the background of limited statistical power, this indicates that the general state of the participants during the intervention did not considerably change. As all participants were in a chronic stage of their iSCI, this result was anticipated.

Statistically, we could also not detect any carry-over or treatment by period interaction. This means that either there is no carry-over, which is very unlikely since the intermediate measurement results differed from baseline measurement results, meaning that “baseline” values before the second intervention were different from baseline measures before the first intervention; or it means that carry-over was comparable for both interventions, and, therefore, insignificant for the interpretation of the achieved results.

The results of the follow-up measurement were double-edged. On the one hand, there were only a few outcome measures that improved over the course of the whole study (from baseline to follow-up). On the other hand, no outcome measure got worse from the end of interventions to follow-up. This would be especially valuable for those outcome measures that improved during the training phase, which are outcomes reflecting increased motor capacity and performance (10MWT at preferred speed, LEMS and SCIM). However, the inability to detect clear differences could be due to the low power of this study. Please note that the follow-up results do not allow a differentiation between the 2 interventions.

### Pain assessments

Overall, both interventions led to an alleviation of pain intensity over time, reflected in steadily decreasing VAS-scores before and after training. This has already been shown for strength training interventions [58] and is herewith presented for the first time for RAGT. If pain intensity was averaged over all sessions and subjects (RAGT: before training: 28.6 ± 3.0% and after training: 24.1 ± 3.3%; strength training: before training: 29.6 ± 2.6% and after training: 22.8 ± 3.6%), this equaled in a mean short-term pain reduction of 15-23%. It must be noted though that in this gait-unrelated outcome measure, strength training seems to have had a larger impact compared to RAGT as there was a significant difference between the changes in scores of mean pain intensity. Nevertheless, we can conclude that neither strength training nor RAGT worsened the general perception of pain. This is particularly important for the Lokomat as it obviously can be adapted to the physiology of the users without overstraining their musculoskeletal system.

We did not investigate pain-relieving effects within a specific type of pain because we had only a small number of subjects, and some of these subjects experienced both musculoskeletal and neuropathic pain. In our opinion, it might be less relevant as there is evidence that physical activity can have positive effects on neuropathic as well as on musculoskeletal pain. With respect to neuropathic pain, several studies suggest that physical activity could have a positive influence on impaired sensory function [14,59-61]. It has been shown in animals that treadmill running has positive effects on nerve regeneration and functional recovery after peripheral nerve injuries [62,63], which are known to cause neuropathic pain [64]. Further literature shows that physical activity can also reduce musculoskeletal pain [65,66].

### Limitations

Sample sizes are known to be rather small in exercise training studies involving subjects with chronic spinal cord injury [67]. This study unfortunately is no exception. Larger sample sizes would have led to greater statistical power. Additionally, due to the high number of (exploratory) outcome measures, this study is also prone to type I and type II errors. Generalizability of the findings is limited to people with the narrowly defined criteria applied in this study and as all participants agreed to take part in this intensive training program, we cannot exclude a motivational bias. In anticipation of the low number of participants, we chose a cross-over design. This has the advantage that each participant acts as its own control, which is specifically valuable in a group with high inter-individual variation. However, there is the disadvantage of possible carry-over. Carry-over and treatment by period interaction are generally thought to be hard to objectify in training studies (especially in this study with its small statistical power) and are highly inter-individual. Therefore, we abstained from introducing a wash-out period, which reduced the load for participants, but this might have also influenced the results. The absolute effectiveness of the interventions could be overestimated, as repeated exposure to the testing protocol or natural recovery over time might have induced functional improvements, despite including patients with a chronic iSCI only.

We did not blind the assessors for the treatment of the participants. However, all 10MWTs were recorded on video and a blinded assessor scored the videotaped 10MWTs. The un-blinded original scores and the blinded videotaped tests matched excellently (linear regression analysis: y = 0.98x + 0.009 m/s; R^2^ = 0.99). Nevertheless, we cannot exclude assessor-bias for other outcome measures.

It might be that the participants in this study were clinically too good for the Lokomat. However, we tried to accommodate this flaw by applying different training modes, and there were only 2 participants, who were able to acceptably manage the most difficult training mode. Nevertheless, strength training is customizable to a much higher extent than RAGT and the therapist can specifically address weaknesses that result in straight improvements of function.

Participants did not perform other physical exercises besides the interventions within this study. However, all but one participant (P05) were community ambulators (with assistive devices) and ambulatory activity besides our trainings was not monitored.

The average intensity of pain in this sample varied much, and it remains speculative whether RAGT could alleviate pain more in participants who experience weaker or in those with stronger pain. While one participant in our study with strong pain profited from the training (P04), the participant who reported the highest pain intensity (P09) did not show any change in pain intensity due to the training.

Optimal training dosage still is unknown. The number of training sessions and the duration of each training session were chosen according to clinical experience and no participant complained about physical overload.

At last, during RAGT we did not focus on specific aims. Usually, we tried to let the participants walk as fast as possible, but as in clinical application, we also focused on minimizing levels of robotic assistance and bodyweight support.

## Conclusion

Task-specific RAGT was not better than lower extremity strength training at improving walking performance in patients with chronic iSCI who depended on walking assistance. In fact, maximum walking speed improved more after strength training compared to RAGT. However, due to the low number of participants, statistical power was limited.

## Abbreviations

10MWT: 10 m walk test; AIS: ASIA impairment scale; ASIA: American Spinal Injury Association; BBS: Berg balance scale; BWSTT: Body weight-supported treadmill training; CI: Confidence interval; CONSORT: Consolidated standards of reporting trials; FES-I: Falls efficacy scale - International version; FET: Figure Eight Test; ISCI: Incomplete spinal cord injury; LEMS: Lower extremity motor score; PCI: Physiological cost index; RAGT: Robot-assisted gait training; SCIM: Spinal cord independence measure; UEMS: upper extremity motor score; VAS: Visual analog scale; WISCI: Walking index for spinal cord injury.

## Competing interests

The author’s declare that they have no competing interests.

## Authors’ contributions

RL participated in the design of the study and coordinated it; he conducted all trainings and assessments, performed data and statistical analyses and drafted the manuscript. HvH conceived of the study, participated in its design, performed statistical analyses and helped to draft the manuscript. Both authors read and approved the final manuscript.
